# Hepatic Adaptation Compensates Inactivation of Intestinal Arginine Biosynthesis in Suckling Mice

**DOI:** 10.1371/journal.pone.0067021

**Published:** 2013-06-13

**Authors:** Vincent Marion, Selvakumari Sankaranarayanan, Chiel de Theije, Paul van Dijk, Theo B. M. Hakvoort, Wouter H. Lamers, Eleonore S. Köhler

**Affiliations:** 1 Department of Anatomy & Embryology, Maastricht University, Maastricht, The Netherlands; 2 Laboratoire de Génetique Médicale, Institut National de la Santé et de la Recherche Médicale (INSERM) U1112, Strasbourg Cedex, France; 3 Tytgat Institute for Liver and Gastrointestinal Research, Academic Medical Center University of Amsterdam, Amsterdam, The Netherlands; National Institute of Agronomic Research, France

## Abstract

Suckling mammals, including mice, differ from adults in the abundant expression of enzymes that synthesize arginine from citrulline in their enterocytes. To investigate the importance of the small-intestinal arginine synthesis for whole-body arginine production in suckling mice, we floxed exon 13 of the *argininosuccinate synthetase* (*Ass*) gene, which codes for a key enzyme in arginine biosynthesis, and specifically and completely ablated *Ass* in enterocytes by crossing *Ass* ^*fl*^ and *Villin-Cre* mice. Unexpectedly, *Ass* ^*fl/fl*^ */VilCre* ^*tg/-*^ mice showed no developmental impairments. Amino-acid fluxes across the intestine, liver, and kidneys were calculated after determining the blood flow in the portal vein, and hepatic and renal arteries (86%, 14%, and 33%, respectively, of the transhepatic blood flow in 14-day-old mice). Relative to control mice, citrulline production in the splanchnic region of *Ass* ^*fl/fl*^ */VilCre* ^*tg/-*^ mice doubled, while arginine production was abolished. Furthermore, the net production of arginine and most other amino acids in the liver of suckling control mice declined to naught or even changed to consumption in *Ass* ^*fl/fl*^ */VilCre* ^*tg/-*^ mice, and had, thus, become remarkably similar to that of post-weaning wild-type mice, which no longer express arginine-biosynthesizing enzymes in their small intestine. The adaptive changes in liver function were accompanied by an increased expression of genes involved in arginine metabolism (*Asl*, *Got1*, *Gpt2*, *Glud1*, *Arg1*, and *Arg2*) and transport (*Slc25a13*, *Slc25a15*, and *Slc3a2*), whereas no such changes were found in the intestine. Our findings suggest that the genetic premature deletion of arginine synthesis in enterocytes causes a premature induction of the post-weaning pattern of amino-acid metabolism in the liver.

## Introduction

Arginine is a substrate for the synthesis of nitric oxide, urea, ornithine, citrulline, creatine, agmatine, glutamate and proline [1]. The rate-determining enzyme of arginine synthesis from citrulline and aspartate is argininosuccinate synthetase (ASS) (EC 6.3.4.5) [2]. The endogenous biosynthetic capacity for arginine suffices to meet the daily requirement under normal conditions, but a dietary source of arginine becomes necessary when demand increases under anabolic or catabolic conditions [3–5]. For this reason, arginine is a conditionally essential amino acid.

These general conclusions apply to post-weaning individuals that can adapt their sources of food. However, arginine metabolism in the early postnatal period differs considerably from that in the adult, probably because the rapidly growing suckling animals have no choice of food. Since the supply of arginine via the milk is not sufficient to support optimal protein synthesis [6–8], suckling animals need a higher endogenous capacity for arginine synthesis. The abundant expression of the enzymes necessary for *de novo* arginine biosynthesis in the enterocytes of the small intestine of perinatal rodents [9–11], piglets [12,13] and humans [14] and the absence of cytosolic arginase expression [9,15–18] suggest that the intestine is an important site for early postnatal arginine production in mammals. In fact, it has been demonstrated that the small-intestinal mucosa accounts for ~50% of the endogenous production of arginine in normally fed suckling piglets, while hypoargininemia develops if this source of arginine production is bypassed as during parenteral feeding [19–21]. In addition, we have shown that suckling mice that transgenically overexpress *arginase1* in the small-intestinal enterocytes suffer from deficient hair and muscle growth, and underdevelopment of Peyer’s patches due to impaired B-cell maturation [15,16]. Interestingly, the capacity of the intestine to produce arginine becomes limited to citrulline synthesis in the weaning period, when the expression of argininosuccinate synthetase and lyase in the intestines declines in humans and pigs [14,17,18], and stops in rodents [9]. Although these findings suggest that intestinal arginine biosynthesis is essential for suckling animals, this hypothesis remains to be tested.

To assess to what extent the small-intestinal arginine synthesis is essential for suckling mice, we have eliminated the expression of argininosuccinate synthetase (*Ass*) in the enterocytes, using the Cre-loxP recombinase system. Since the total-body deletion of *Ass* is lethal [22], exon 13 of the *Ass* gene was surrounded by loxP sites. Enterocyte-specific excision of the “floxed” exon was accomplished by breeding *Ass* ^*fl*^ mice with *VilCre* mice [23]. Suckling *Ass* ^*fl/fl*^ */VilCre* ^*tg/-*^ mice (*Ass*-KO/I) were completely devoid of *Ass* expression in their enterocytes, but unexpectedly did not suffer from growth retardation or any other detectable functional impairment. Whereas *Ass* elimination from enterocytes did not cause adaptive changes in gene expression in enterocytes, it did change the expression of several amino acid-metabolizing and transporting genes in the liver with as result that arginine deficiency did not develop. Our findings highlight a homeostatic regulatory interaction of the gut-liver axis with respect to arginine metabolism.

## Methods

### Plasmid construction and recombinant ES cell selection

The mouse *Ass* gene is located on chromosome 2. The targeting vector (14.384 Kb) consisted of the 3' 4.2 Kb of intron 12, a Neo-TK selection cassette flanked by *frt* sites, exon 13 flanked by *loxP* sites and the 5' 3.5 Kb of intron 13 (Figure S1A). The targeting construct was sequence verified with respect to splice junctions, exon 13 and recombinase-recognition sites, digested with AscI and PmeI (introduced by PCR for cloning purposes), and purified by electrophoresis and electro-elution. Twenty-five µg of the targeting fragment was electroporated into the 129P2/OlaHsd-derived mouse ES cell line E14IB10, a subclone of E14 cells [24]. Selection with G418 (200 µg/mL) was started 24 hours after electroporation. Short vector sequences that were left on either end of the targeting construct allowed a PCR-based negative screen against random integrations (Figure S1B, upper panels). Long-distance PCR with an *Ass*-specific primer outside the targeting construct and a *loxP*-specific primer was used to demonstrate homologous recombination at the 3’ side in the remaining clones (Figure S1B, middle panel). Proper recombination of the 5’ end was demonstrated with Southern blotting and probing with a Neo fragment (Figure S1B, lower panel). The Neo-TK cassette was removed by transient transfection with an *FLPe* recombinase expression vector (kindly provided by Dr. Francis Stewart, EMBL, Germany). Selection with ganciclovir (5µM) was started 5 days after electroporation. The recombined allele was again sequence verified (ganciclovir introduced a mutation in 1 of 4 clones tested). Two clones containing 40 chromosomes were selected for blastocyst injection.

### Generation of transgenic mice and husbandry

**Figure 1:**
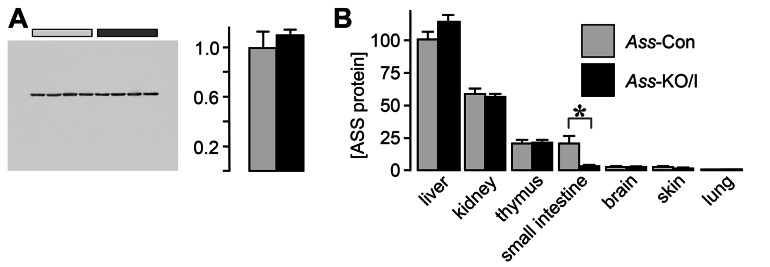
Expression of the *Ass* ^*fl*^ allele is not compromised and allows efficient excision. Panel A: Western blot demonstrating that the *loxP* sites surrounding exon 13 do not affect ASS expression at the protein level (liver extract with 25µg total protein was loaded per lane; quantification of band intensity on right subpanel). Panel B: ASS protein in ND14 *Ass*-KO/I mice is eliminated in the enterocytes of the small intestine only. Note that the residual ASS in the small intestine reflects the presence of the ASS-positive enteric ganglia (Figure 2). Light gray bars indicate *Ass*-Con mice and dark gray bars *Ass*-KO/I mice (N=4 each).

*Ass*-recombinant ES cells were injected into C57BL/6J blastocysts. Chimeric male mice were bred with female 129P2/OlaHsd mice (Harlan, The Netherlands) and with deleter-Cre females on a C57BL/6 background (kindly provided by Dr. Ursula Lichtenberg, University of Cologne, Germany) to delete *Ass* exon 13 in the germ line [25]. Mice were genotyped with primers *Ass-F1* and *Ass-R1* (Table S1 Figure S1C and D, upper subpanel), yielding a 360 bp wild-type allele and a 390 bp floxed allele. The Cre-excised allele (340 bp) was detected by PCR with the primers *Ass-F2* and *Ass-R1* (Figure S1C and D, middle subpanel). To specifically delete the *Ass* ^*fl*^ allele in the enterocytes of the small intestine, mice were crossed with *VilCre* mice [23]. Animals were genotyped for the presence of *VilCre* by PCR with primers *Vil-F* and *Vil-R* (see: Table S1 and Figure S1D, lower subpanel), yielding a 1,100 bp band for *VilCre*-positive animals. All mice used in this study except the controls in Figure 1A had the same genetic background.

Animals were housed in humidity- and temperature-controlled rooms on a 12-hour light/ 12-hour dark cycle with food and water ad libitum. The animal studies were reviewed and approved by the institutional Animal Care and Ethical committee (DEC) of Maastricht University.

### Blood and tissue collection

Animals were sacrificed by decapitation on neonatal days (ND) 14, 18, 21, and 35. Blood samples from the hepatic vein, portal vein, renal vein and left ventricular cavity were taken from 14- and 35-day-old mice under 2% isoflurane (Abbott, # B506)/ 90% O_2_ anesthesia, as described for adult mice [26]. Tissues were fixed in 4% buffered formalin and embedded in Paraplast®.

### Amino-acid concentration

Blood was collected into heparin-coated tubes and centrifuged at 2,000*g for 5 min at 4^°^C. 80 µL of plasma was added to 6.4 mg of lyophilized sulphosalicylic-acid, vortexed and stored at -20^°^C. Amino-acid concentrations were determined by HPLC as described [27]. Amino-acid fluxes were calculated as described [26]. Because only the relative distribution of flows across organs could be measured (next section), fluxes were expressed as the product of the arterio-venous differences in amino-acid concentrations and the relative flow across the respective organs.

### Blood flow distribution using radioactive microspheres

Radioactive microspheres (15 µm diameter), labeled with ^103^Ru, were purchased from PerkinElmer USA (NEM082A100UC). Fourteen-day-old mice (6-8 gram body weight) were anesthetized by intraperitoneal injection of 100 µg/g ketamine (Pfizer) + 20 µg/g xylazine. After opening the thorax through the fourth left intercostal space, 50 µL of a 0.01% Tween-80/saline solution containing 10,000 microspheres (~20 KBq) was injected into the left ventricle. Organs were isolated, transferred to a plastic counting vial containing 2 mL of saline and measured with a gamma counter (Wallac Wizard 1480; PerkinElmer, USA).

### Western blotting and histology

Tissues were crushed in a liquid nitrogen-cooled mortar, sonicated in RIPA buffer (25 mM Tris·HCl(pH=7.6), 150 mM NaCl, 1% NP-40, 1% Na-deoxycholate, 0.1% Na-dodecylsulfate), containing a protease inhibitor cocktail (Complete; Roche, The Netherlands). Twenty-five µg of protein was loaded per lane. Equal protein loading per lane was verified by Ponceau-S staining of the blots prior to incubation with antiserum. ASS was visualized by incubating the blots with rabbit anti-mouse ASS antiserum (1:1,500 [15,16]), followed by alkaline phosphatase-coupled goat anti-rabbit antibody and "Super Signal West Femto Maximum Sensitivity Substrate" (Pierce # 34095).

Duodenal (2 cm distal to the pyloric sphincter) and ileal (2 cm proximal to the ileocecal junction) segments were fixed in ice-cold buffered 4% formaldehyde, embedded in Paraplast®, and sectioned at 7 µm. Goblet cells were visualized with 1% Alcian blue (BDH, The Netherlands) in 3% acetic acid (pH = 2.5) for 30 min. Nuclei were visualized with 1% Nuclear Fast Red (Chroma, Fluka) for 3 min. For immunostaining, sections were subjected to antigen retrieval (10 min at 100°C in 10 mM Na-citrate (pH 6.0)), blocked with Teng-T (10 mM Tris (pH=7.6), 5 mM EDTA, 150 mM NaCl, 0.25% gelatin, 0.05% Tween-20) /10% normal goat serum (NGS, Gibco, The Netherlands) for 30 min, and incubated overnight in a 1:1,000 dilution of the ASS antibody [15,16] in Teng-T/10% NGS. After washing with PBS and another blocking step with Teng-T /10% NGS for 15 min, sections were incubated with goat anti-rabbit antiserum (Sigma A3687; diluted 1:200 in Teng-T /10% NGS) for 90 min. After washing, the slides were incubated with nitroblue tetrazolium chloride/5-bromo-4-chloro-3-indolyl phosphate (NBT/BCIP; Roche 169471) at 37^°^C until staining reached an optical density not exceeding 0.8.

### Visualization of Peyer’s patches

The small intestine was immersed for 5 min in 10% acetic acid (v/v) before fixation in 10% neutral-buffered formalin, as described previously [16].

### PCR assays

RNA was extracted from cells and tissues with guanidinium thiocyanate (TRI Reagent, Sigma). RNA integrity was assessed by denaturing gel electrophoresis and RNA concentration with a NanoDrop-ND1000 spectrophotometer (Isogen Life Sciences). One µg of total RNA was reverse transcribed with the iScript cDNA synthesis kit (Bio-Rad). The cDNA preparation was diluted 1:10 (v/v) with milliQ water prior to addition to the PCR mixtures.

#### Quantitative PCR

PCR was performed using iQTM SYBR® Green Supermix (Biorad 170-8882) in an iQ5 real-time PCR detection system (Biorad). The extension temperature was 60^°^C for all primer pairs. cDNA samples were diluted 60-fold before use for mRNAs and 600-fold for 18S rRNA. Primary fluorescent data were exported and analyzed using the Lin-RegPCR program [28]. All primers (Table S1) were obtained from Genosys (Sigma).

#### PCR array

TaqMan® OpenArray® Real-Time PCR Plates containing validated primers for mRNAs of 42 different genes involved in arginine metabolism or transport (Table S2) were ordered from Life Technologies (Saint-Aubin, France). The PCR assays were run according to the “rapid” program in an ABI cycler.

### Statistics

Between-session variation in replicate experiments was corrected using “Factor Correction” [29]. All quantitative values are presented as means ± SEM. Statistical differences for the amino acids fluxes between genotypes were based on homogeneity of variances between the blood sources per genotype followed by one-way analysis of variances using known means and standard deviations. P-values were calculated through multiple comparisons [30].

## Results

### Characterization of Ass^fl^ mice

To study the function of ASS in the enterocytes of the small intestine, we generated a conditional *Ass* knockout, in which the critical exon 13 is flanked by *loxP* sites (Figure S1). Mice with *Ass* ^*‑/fl*^ and *Ass* ^*fl/fl*^ genotypes were healthy and fertile. In agreement, Figure 1A shows that flanking exon 13 with *loxP* sites did not affect ASS protein content in the liver, an organ with very high *Ass* expression (Figure 1B). To test if deletion of exon 13 in the mouse was as effective (lethal) as deletion of exon 4 [22], we interbred *Ass* ^*+/-*^ mice (generated by crossing *Ass* ^*+/fl*^ mice with deleter-Cre mice [25]). Figure S2A demonstrates that none of the *Ass* exon13-deficient *Ass* ^*-/-*^ neonates survived longer than 1 day after birth, as was found for the constitutive *Ass* exon4-deficient mice [22]. Together, these data show that mice harboring the *Ass* ^*fl*^ allele are a good model to investigate the effects of tissue-specific *Ass* deletions. *Ass* ^*fl/fl*^ */VilCre* ^*-/-*^ mice are indicated as *Ass*-Con and *Ass* ^*fl/fl*^ */VilCre* ^*tg/-*^ as *Ass*-KO/I.

### VilCre efficiently and specifically eliminates Ass in the enterocytes

**Figure 2:**
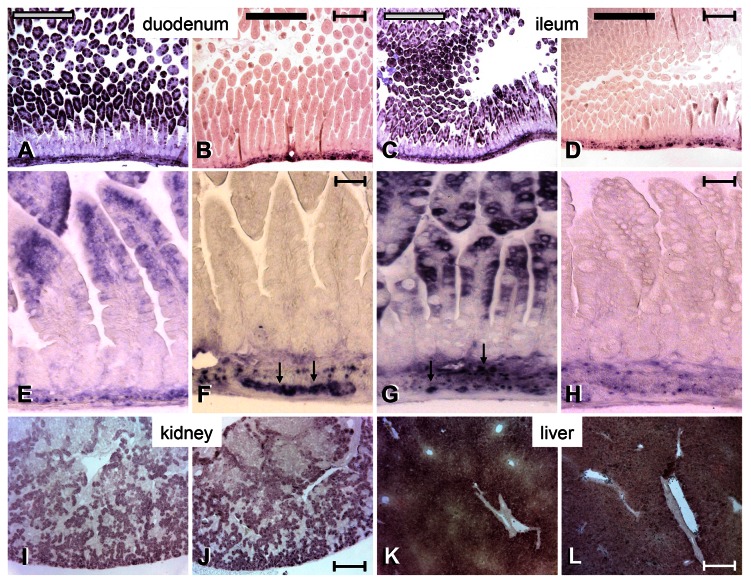
Expression of ASS in the small intestine, kidney and liver of ND14 mice. ASS protein in the duodenum (**panels A, B, E, F**) and ileum (**panels C, D, G, H**) of *Ass*-Con or *Ass*-KO/I animals is visualized by semiquantitative immunohistochemistry. The second row of panels shows magnifications of the top row. Note that expression of ASS in *Ass*-Con mice is limited to the top portion of the villus. Further note that the elimination of *Ass* expression is complete in the enterocytes of *Ass*-KO/I mice, but unaffected in their enteric ganglia (arrows). The bottom panels show unperturbed ASS protein in the S3 segment of the proximal tubule of the kidney (J, K) and in the periportal region of the liver (L, M) of *Ass*-KO/I mice. Gray bars identify *Ass*-Con and black bars *Ass*-KO/I mice. Bars: 8 µm (overviews) and 1 µm (magnifications).

To evaluate the development of the small intestine in the absence of *Ass* expression in the enterocytes, sections of the intestines of ND14, ND18, and ND21 mice were stained with haematoxylin and eosin or with Alcian blue and Nuclear Fast Red. No qualitative differences in the architecture of the duodenum and ileum were found between *Ass*-Con and *Ass*-KO/I mice at any of the ages investigated (not shown). Staining the duodenum and the ileum for the presence of ASS revealed a complete elimination of ASS in the enterocytes of *Ass*-KO/I mice only (Figure 2A–H), whereas ASS in the enteric ganglia remained unaffected. Although both *Ass* and *Vil* are also expressed in the proximal tubules of the kidney [31,32], the *VilCre* transgene is not [23]. Accordingly, we did not observe the disappearance of ASS protein in the kidney (Figure 1B Figure 2I,J). Both *Ass* and *Vil* are also expressed in the epidermis [33,34]. Although ASS protein content in the skin of *Ass* ^*fl/fl*^ */VilCre* ^*tg/-*^ mice was lower than in *Ass* ^*fl/fl*^ */VilCre* ^*-/-*^ mice (Figure 1B), the difference was not significant.

### Development in the absence of Ass expression in the enterocytes

F/A2 mice, which have no net production of arginine in their enterocytes due to the transgenic over-expression of *arginase1* in these cells, suffer from impaired hair and muscle growth and underdevelopment of Peyer’s patches due to impaired B-cell development during the suckling period [15,16]. Despite the complete elimination of *Ass* expression in their enterocytes, *Ass*-KO/I mice show a normal increase in body weight (Figure S2B), hair growth (Figure S2C), and development of Peyer’s patches (Figure S2D).

### Changes in plasma amino-acid concentrations in the absence of Ass expression in enterocytes

**Figure 3:**
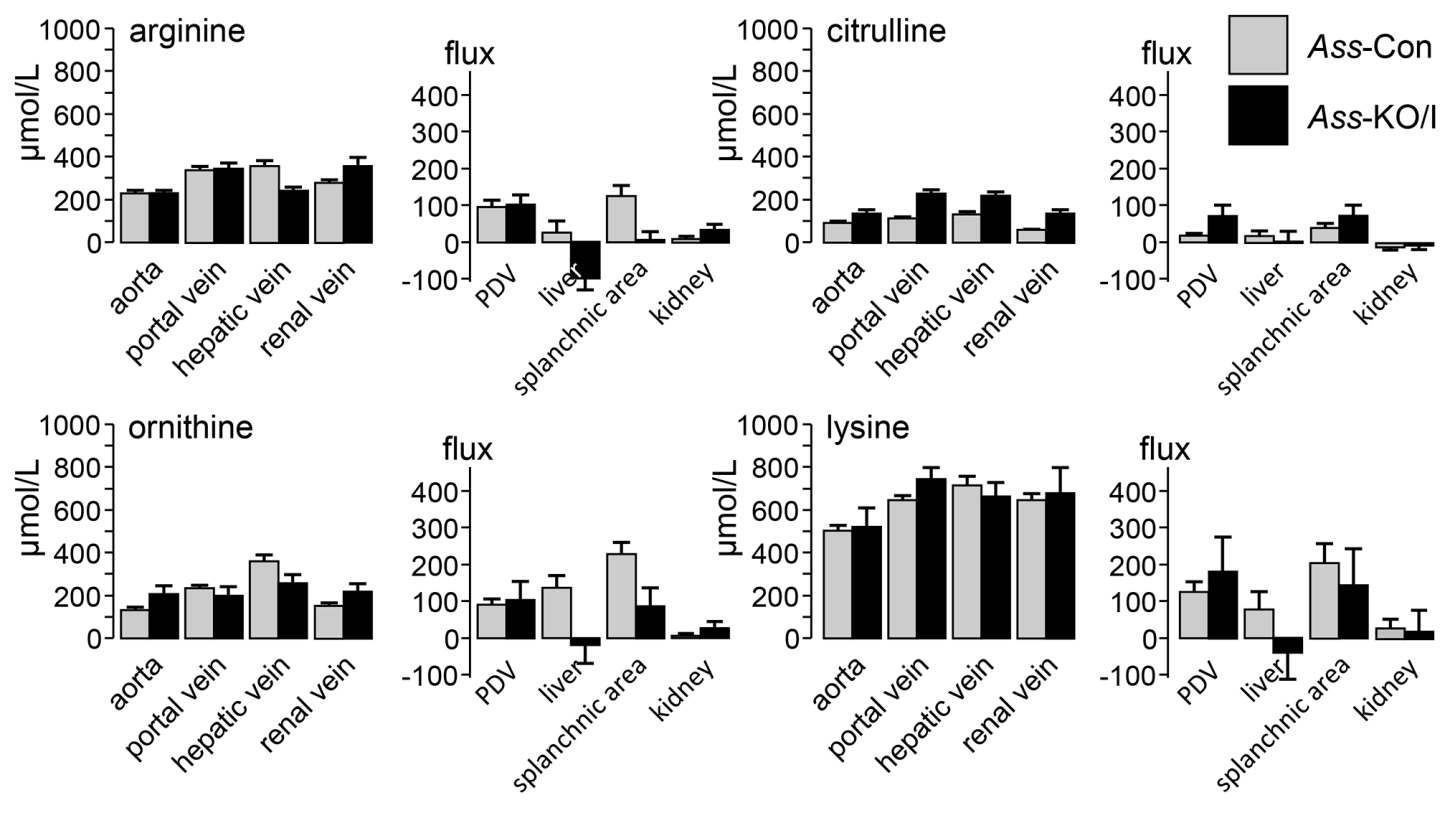
Circulating levels and fluxes of arginine, citrulline, ornithine, and lysine in ND14 mice. **Left panels**: Circulating concentrations of the indicated amino acids in *Ass*-Con (light gray) and *Ass*-KO/I (black) mice. The source of the blood is indicated. **Right panels**: Production or consumption of the indicated amino acids across the splanchnic region, portal-drained viscera (PDV) and liver.

**Figure 4:**
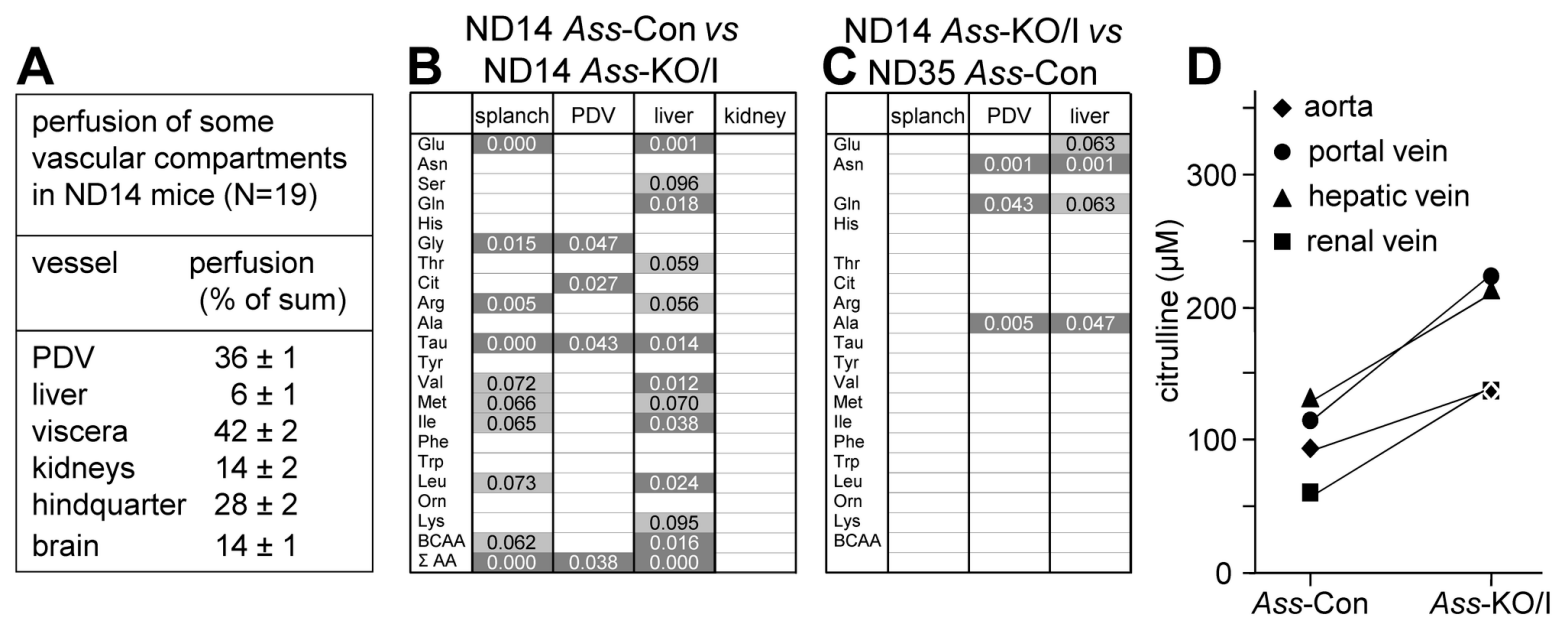
Regional perfusion, differences in amino-acid fluxes and citrulline concentration in ND14 *Ass*-Con and *Ass*-KO/I mice. Panel **A**: Blood flow in portal vein (“Portal Drained Viscera”), hepatic artery (liver), hepatic vein (viscera), renal arteries (kidneys), femoral arteries (hindquarter) and brain as assessed by microsphere distribution after intracardiac injection. Panel **B**: Significant differences in the flux of amino acids across the splanchnic region, portal drained viscera, liver, and kidneys of ND14 *Ass*-KO/I compared to ND14 *Ass*-Con mice. **C**: Significant differences in the flux of amino acids across the splanchnic region, portal drained viscera and liver of ND14 *Ass*-KO/I compared to ND35 *Ass*-Con mice. In panels B and C, dark gray fields represent differences with P ≤ 0.05. Differences with 0.05 ≤ P ≤ 0.10 (light gray) are also shown, because the power of our calculations was weakened by the fact that we could take only one blood sample from a vessel in each ND14 pup. Panel **D**: Average concentration of citrulline in the respective vessels (cf. also Table S3).

Circulating amino acids in arterial, portal, hepatic and renal blood of *Ass*-Con and *Ass*-KO/I mice were determined at ND14 and ND35 (Table S3), with those of arginine, citrulline, ornithine and lysine in ND14 shown in Figure 3A–D. Lysine was included, because it is, like arginine, positively charged and transported by the same transporters, so that differences in the prevalence of arginine would probably be reflected in those of lysine [35–37]. Compared to *Ass*-Con mice, the circulating concentration of arginine in plasma of *Ass*-KO/I mice did not change significantly in the aorta and portal vein, but decreased to ~65% in the hepatic vein and increased ~35% in the renal vein (Figure 3A). Circulating concentrations of the arginine precursor citrulline changed more profoundly, being ~1.5-fold higher in the aorta and hepatic vein and ~2-fold higher in the portal and renal veins of *Ass*-KO/I than of *Ass*-Con mice (Figures 3B and 4D). The concentration of ornithine was decreased in the hepatic vein and increased in the renal vein of *Ass*-KO/I compared to *Ass*-Con mice (Figure 3C). Plasma lysine concentrations were not different between control and experimental animals (Figure 3D). With respect to the other amino acids and compared to *Ass*-Con mice, glutamate, alanine and taurine increased in the aorta, glutamate, glutamine, threonine, alanine, taurine, valine, and leucine increased in the portal vein, and alanine, taurine, valine, isoleucine, leucine in the renal vein of *Ass*-KO/I mice, whereas glutamate, valine, isoleucine, tryptophan, and leucine decreased in the hepatic vein (Table S3). In agreement with these findings, the sum of amino acids was higher in the aorta, and portal and renal veins, and lower in the hepatic vein of *Ass*-KO/I than of *Ass*-Con mice. Strikingly, the data of the experimental group were more heterogeneous than those of the control group.

### Changes in amino-acid fluxes in the absence of ASS in enterocytes

The uptake or release of amino acids across the intestine, liver and kidneys of suckling ND14 mice was determined by multiplying the veno-arterial concentration differences and the blood flow across these organs. To measure the blood flow across the organs, we determined the distribution of radioactive microspheres injected into the left ventricular cavity (Figure 4A). Since we were not able to harvest reference samples from the abdominal aorta of these small mice (~8 gram), only the relative distribution of the cardiac output across organs and, hence, the metabolic fluxes in the portal drained viscera, liver, and kidneys could be calculated. At ND14, ~86% of the transhepatic blood flow originated in the portal vein and ~14% in the hepatic artery, while blood flow through the kidneys amounted to ~33% of that through the liver, without significant differences between genotypes. Relative to *Ass*-Con mice, the production of arginine, ornithine, and lysine in the portal-drained viscera had not changed in *Ass*-KO/I mice, but the liver had become an arginine- and lysine-consuming organ (significance at the trend level), so that the contribution of the splanchnic region to systemic arginine even became non-existent (Figure 3A, right panel). Arginine production in the kidney had more than doubled, but this compensated only ~25% of the decrease in arginine production in the splanchnic area. Citrulline was the only amino acid with an increased (~4-fold) production in the portal drained viscera of *Ass*-KO/I mice, due to the absence of ASS (Figure 3B, right panel). The trends of arginine and lysine were followed by the other amino acids: only the production of glycine and taurine decreased significantly in the portal drained viscera, whereas the liver changed from production to uptake for glutamate, glutamine, taurine, valine, isoleucine, leucine, and the sum of all amino acids (Table S3 Figure 4B). Furthermore, a trend with the same effect was seen for serine, threonine, and methionine. These data therefore show that deletion of *Ass* from the enterocytes of the small intestine has little effect on the small intestine itself, but exerts a major effect on the liver, significantly decreasing the hepatic production of 6 amino acids (glutamate, glutamine, taurine, valine, isoleucine, and leucine) and that of 5 more amino acids (serine, threonine, arginine, methionine, and lysine) at the trend level, that is, changing the liver from an amino acid-producing into an amino acid-consuming organ. Due to the relatively small blood flow through the kidneys (~33% of that through the splanchnic region), the contribution of the kidney to amino-acid metabolism was still minor at 14 days after birth (Table S3) and not different between controls and knockouts (Figure 4B).

### Changes in plasma amino-acid concentrations and fluxes in the absence of ASS in enterocytes

We also measured amino-acid concentrations in arterial, portal and hepatic venous blood of post-weaning (35-days old) mice, that is, when *Ass* is no longer expressed in the small intestine of mice. Table S4A shows that, at this age, *Ass*-KO/I mice no longer differed from *Ass*-Con mice in the concentration of blood amino-acid concentrations. In agreement, the fluxes of amino acids across the portal-drained viscera and liver also differed no longer (Table S4B). Furthermore, it should be noted that, with the exception of glutamine, the liver had become an amino-acid consuming organ in both *Ass*-KO/I and *Ass*-Con mice. Compared to ND14 Ass-KO/I mice, ND35 (Figure 4C) mice merely differed in a further increase in the hepatic consumption of glutamate, asparagine, and alanine, and in the production of glutamine, probably reflecting the detoxification of ammonia in pericentral hepatocytes.

### Changes in gene expression in small intestine and liver in the absence of ASS in enterocytes

**Figure 5:**
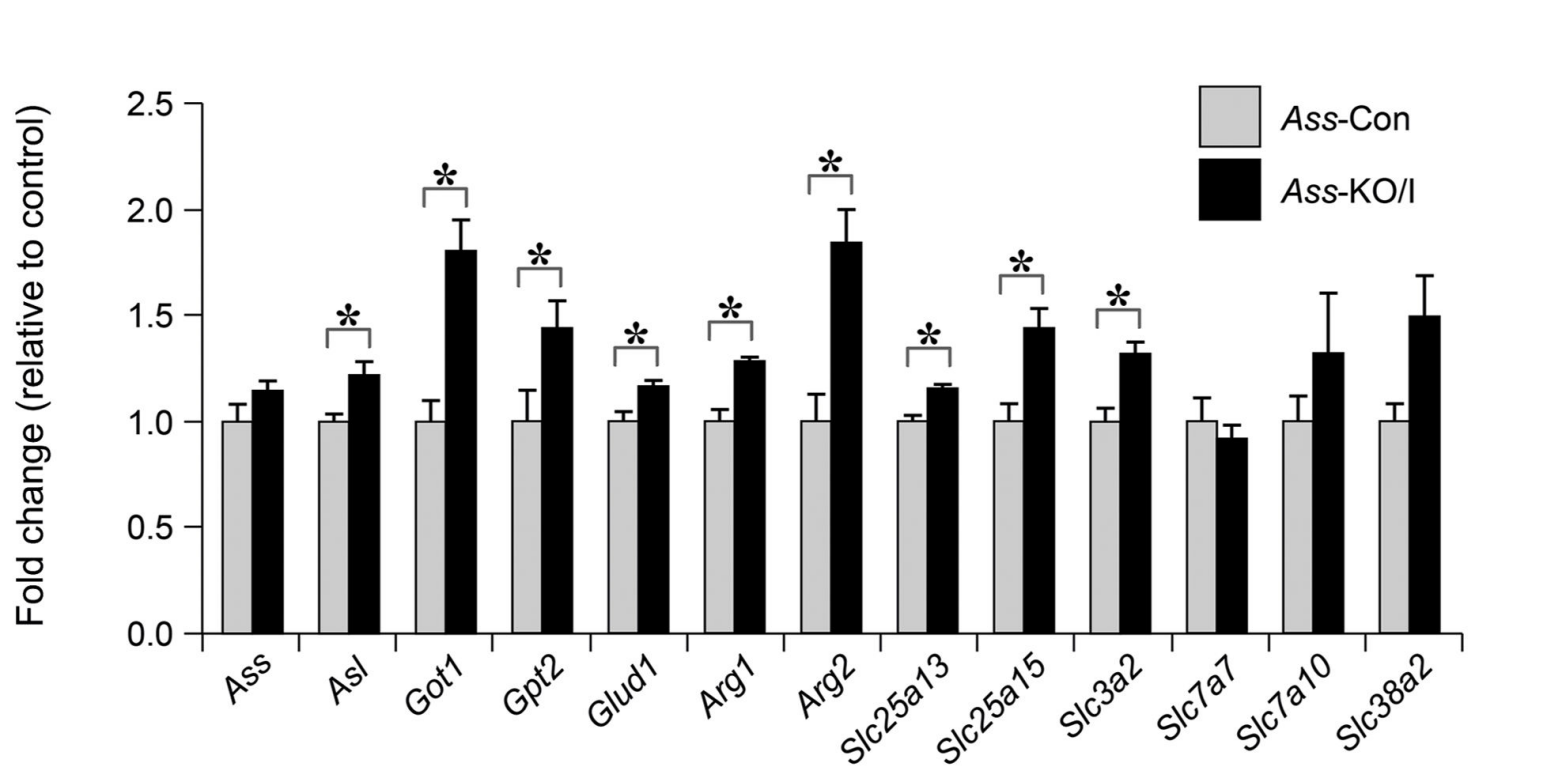
Adaptive changes in arginine transporting or metabolizing genes in the liver of *Ass*-KO/I mice. The 13 genes were selected from a panel of 45 genes (Table S2) and tested on cDNA preparations from 8 *Ass*-Con (light-gray bars) and 8 *Ass*-KO/I mice (black bars). *: P<0.05 between *Ass-*Con and *Ass-*KO/I.

We designed a PCR array to measure the expression of 45 genes involved in arginine metabolism and arginine, citrulline, or ornithine transport (Table S2). These arrays were tested on 3 small intestines and 3 livers of *Ass*-Con and of *Ass*-KO/I mice. Of these, none except *Ass* differed in intestinal expression between ND14 *Ass*-Con and *Ass*-KO/I mice. In 3 ND14 *Ass-*KO/I livers, however, the expression of *Ass, Asl, Got1, Gpt2, Glud1, Arg1, Arg2, Slc3a2, Slc7a7, Slc7a10, Slc25a13, Slc25a15*, and *Slc38a2* was increased. These results were then confirmed by standard qPCR assays in 5 additional livers of *Ass*-Con and of *Ass*-KO/I mice (Figure 5). In this assay, significant upregulation of gene expression was found for *Asl, Got1, Gpt2, Arg1, Arg2, Slc3a2, Slc25a15*, and *Slc38a2*, demonstrating that the liver adapts to the loss of *Ass* expression in the gut. These 9 genes encode enzymes (*Asl*, *Got1*, *Gpt2*, *Glud1*, *Arg1*, and *Arg2*) and transporters (*Slc25a13* (aspartate/glutamate carrier), *Slc25a15* (ornithine transporter), and *Slc3a2* (light subunit of LAT1 transporter)) that are all directly linked to amino-acid catabolism and urea synthesis. 

## Discussion

Since milk is relatively deficient in arginine [6–8], endogenous synthesis is necessary to ensure optimal growth and development of neonates during the suckling period. Studies in mice, pigs and humans have identified the enterocytes of the small intestine as a site with a potentially large biosynthetic capacity for arginine that disappears after weaning [9–21,38]. Interestingly, our findings demonstrate that this temporary function of the small intestine is dispensable in mice and compensated for by the liver and, to a lesser extent, the kidneys. Indeed, the genetic ablation of *Ass* from enterocytes did not depress the arterial concentration of arginine. Whereas the change in intestinal function was not associated with a change in gene expression, that of the liver was mediated by a wide-spread upregulation of the expression of genes involved in amino-acid metabolism and transport.

### Comparison of Ass-KO/I mice with other arginine-deficient mouse models

Elimination of *Ass* exon 13 by *VilCre*-mediated excision in the small-intestinal enterocytes resulted in undetectable levels of ASS protein in the targeted cells only (Figures 1B and 2). ASS in other tissues, such as the enteric ganglia, kidney tubules, and hepatocytes remained unchanged (Figures 1B and 2). Suckling *Ass*-KO/I mice showed, nevertheless, none of the features of suckling “F/A2” mice, in which intestinal arginine synthesis was annulled by transgenic overexpression of *arginase1* in the enterocytes [15,16]. F/A2 mice suffer from a selective decline in circulating arginine, deficient hair and muscle growth, and underdevelopment of Peyer’s patches. The discrepancy of the phenotypes of *Ass*-KO/I and F/A2 mice suggests that overexpression of *arginase1* causes the gut to function additionally as a sink for circulating and dietary arginine that passes the intestine. Clearly, the capacity of this sink exceeded the endogenous arginine production. The absolute requirement for arginine supplementation of neonatal mice that are constitutively deficient for *ornithine aminotransferase* (*Oat*) [39] also contrasts with the absence of a phenotype in *Ass*-KO/I mice. OAT converts glutamate to ornithine, is expressed at ~5-fold higher levels in the suckling than the weaned small intestine and results in a severe deficiency of ornithine, citrulline and arginine if absent in the neonatal period [39]. Similarly, the *Spf* mutation in ornithine transcarbamoylase (Otc), which severely decreases its capacity to convert ornithine into citrulline, causes a deficiency in circulating citrulline and arginine levels in suckling mice [40]. In aggregate, these mouse models demonstrate that intestinal citrulline is crucial to ensure optimal development whereas intestinal arginine production is, against expectations, expendable to maintain normal circulating arginine levels prior to weaning.

### The metabolic consequences of intestinal deletion of Ass

The absence of hypoargininemia or any other detectable sign of arginine-dependent deficiency, therefore, disproved our hypothesis that intestinal arginine synthesis was necessary to supplement endogenous and dietary arginine sources to sustain growth in the suckling. Only for citrulline, we found a significant ~1.8-fold increase in the circulating concentration in *Ass*-KO/I mice. The assessment of the fluxes of amino acids across the portal drained viscera, liver and kidney in the suckling mice (Table S3B) was more laborious than that in adult mice, because only a single vessel could be cannulated per mouse. Furthermore, we could only determine the relative contribution of the intestine and the liver to splanchnic perfusion, because it was not possible to take a reference sample in the suckling mice to determine cardiac output. Our measurements of the blood flow showed, nevertheless, that the contribution of the hepatic artery to liver perfusion (~14%) was similar to that found in adult mice ([41]; Hakvoort & Lamers, unpublished observations). Furthermore, the perfusion of the ND14 kidney was, relative to adult mice, still small (30-35% instead of 70-90% of the total hepatic blood flow ([41]; Hakvoort & Lamers, unpublished). The contribution of the hepatic artery to liver perfusion in mice is, therefore, only ~50% of that in humans. Apart from a ~4-fold increase in citrulline and similarly pronounced decreases in glycine and taurine production, none of the amino-acid fluxes across the portal drained viscera changed, but the flux of 11 individual amino acids and the sum of all amino acids across the liver all changed from production to consumption. It should be emphasized that it is the veno-arterial concentration difference of metabolites and not the blood flow that determines whether an organ produces (positive difference) or consumes (negative difference) metabolites. Despite the sizable concentration of ASS in the kidneys, these organs did not contribute to the metabolic adaptation of *Ass*-KO/I mice. These findings made us investigate the liver as the organ that compensated for the lack of *Ass* expression in the enterocytes.

### The liver exerts its homeostatic function with adaptive changes in gene expression

Whereas no changes in gene expression could be detected in suckling intestines from which the capacity to produce arginine had been removed, extensive adaptive changes in gene expression were concurrently induced in the liver, which resulted in the maintenance of a normal plasma arginine concentration in the systemic circulation. Such a homeostatic function of the liver with respect to circulating arginine concentrations was also noticed in metabolic studies of suckling and adolescent pigs. In suckling piglets, plasma arginine concentrations remained similar irrespective of whether they were fed via the gut, the portal vein or the caval vein, even though endogenous arginine synthesis is high only in enterically fed animals [42]. Similarly, arginine-enriched foods, such as soy, increased the concentration of arginine in the portal vein of 2-months old pigs, but this dietary “excess” was neutralized by uptake in the liver [43]. In fact, the switch of liver function from that of amino-acid production to that of amino-acid consumption decreased the net production of amino-acids in the splanchnic region of fed suckling *Ass*-KO/I mice to ~35% of that of their control littermates. Our data show that this switch in liver function was mediated, among others, by widespread increases in expression of arginine-metabolizing and transporting genes. Although the signals that mediate these changes in gene expression in the liver remain to be identified, it is of note that the function of the liver of ND14 *Ass*-KO/I and ND35 *Ass*-Con mice was remarkably similar in the sense that the liver was an amino-acid consuming organ in the absence of arginine biosynthesis in the intestine. The major differences in hepatic function between ND14 *Ass*-KO/I and ND35 *Ass*-Con mice were that the consumption of 3 amino acids, of which alanine was quantitatively the most important, was higher in ND35 mice, and that the production of glutamine had increased. In all likelihood, hepatic glutamine production after weaning reflects the detoxification of ammonia in pericentral hepatocytes. This finding implies that the elimination of arginine synthesis from enterocytes, as occurs naturally after weaning, also induces a post-weaning pattern of amino-acid metabolism in the liver if this capacity is eliminated genetically before birth.

### Function of intestinal arginine biosynthesis in neonatal mammals

Although the analysis of genetic mouse models demonstrated that intestinal citrulline synthesis is necessary to maintain normal circulating arginine levels during the suckling period, studies in piglets [20] and (premature) infants [44,45] show that total parenteral nutrition, a condition that is associated with a pronounced decrease in intestinal arginine synthesis in suckling piglets [20], is associated with hypoargininemia and hyperammonemia. These data indicate that intestinal arginine synthesis has a function in piglets and (premature) infants. Extra arginine stimulates, for instance, the synthesis of N-acetylglutamate, a required co-factor for carbamoylphosphate synthetase-1, in enterocytes and hepatocytes [46]. In agreement, some polymorphisms in the carbamoylphosphate synthetase (*Cps1*) gene [47] and hypoargininemia [48,49] predispose premature infants to the development of necrotizing enterocolitis. Similarly, parenteral feeding of premature piglets, which annuls enteral arginine synthesis [9–21,38,42], sensitizes them to a condition that closely resembles necrotizing enterocolitis [50]. To the best of our knowledge, however, a condition resembling necrotizing enterocolitis is not yet described in neonatal mice. In fact, even though the circulating arginine concentration in neonatal F/A2 mice was only ~30% of control values [15,16,51], we never observed any sign of ischemic lesions in their intestines. Apparently, enteric arginine synthesis is less vital to rodents, possibly because neonatal mice and rats hardly produce urea [52].

## Supporting Information

Figure S1 Production and molecular analysis of conditional *Ass* knockout mice. Panel **A**: **S**chematic representation of the *Ass*-targeting construct (AscI – PmeI). The AscI and PmeI sites flanking the construct (14.38 Kb) were introduced by PCR. The XmnI and EcoRI sites upstream from the recombination arms of the targeting construct were used for Southern-blot characterization. Panel **B**: Identification of successfully targeted ES cells. Top subpanel: PCR amplification of vector sequences downstream of AscI (left) or upstream of PmeI (right) identify clones that carried random integrations of the targeting vector. Middle subpanel: Long PCR to demonstrate homologous recombination on 3’ side with primer in loxP site and primer downstream of PmeI, yielding a band of 3.7kb. Bottom subpanel: Southern blot of ES cell DNA with proper 3’ homologous recombination after digestion with XmnI and EcoRI. Two fragments of the expected size (8.663kb and 10.158kb) were detected with the internal Neo probe. Panel **C**: Wild-type (*Ass^+^*) exon-13 allele, floxed (*Ass^fl^*) allele, and *Ass*-deficient (*Ass^‑^*) allele, together with remnant *frt* and *loxP* sites. F1, F2, and R1 indicate the position of the primers used to identify these genotypes (for sequences, see Table S1). Panel **D**: Mouse genotyping. Top subpanel: The F1 and R1 primer pair yield a band of 360 bp for the *Ass* ^*+*^ allele and 390 bp for the *Ass* ^*fl*^ allele. Middle subpanel: The F2 and R1 primer pair yield a band of 340 bp for the *Ass* ^‑^ allele. Bottom subpanel: The presence of the *VilCre* construct was detected with primer pair Vil-F and Vil-R, giving a band of ~1,100 bp.

Figure S2 Biological effects of deletion of exon 13 of the mouse *Ass* gene. Panel **A**: Cre-mediated homozygous germ-line elimination of exon 13 causes neonatal death, as was previously shown for constitutive elimination of exon 4 [44,45]. **Panels B–D**: *Ass*-KO/I mice grow normally during the first 4 postnatal weeks (B, N = 8 for the wild type and the knockout mice), have normal hair growth (ND14; C) and normal development of Peyer’s patches in the small intestine (ND14; D). Light gray bars indicate *Ass*-Con and black symbols *Ass*-KO/I mice.

Table S1 Primer sequences, annealing temperatures and expected PCR-product lengths.

Table S2 Genes tested for expression in the small intestine and liver of ND14 *Ass*-Con and *Ass*-KO/I mice.

Table S3 Plasma amino-acid concentrations and fluxes in ND14 Ass-Con and Ass-KO/I mice. Panel A: Amino acid concentrations in plasma (μM; mean ± SEM). Panel **B**: Amino-acid fluxes (mean ± SEM) of Ass-Con and Ass-KO/I mice are expressed in arbitrary units (arterio-venous difference in concentration * relative flow across the respective organs). Production is indicated in black and consumption in red numbers. Statistical evaluation was performed by ANOVA (see Figure 4B).

Table S4 Plasma amino-acid concentrations and fluxes in ND35 Ass-Con and Ass-KO/I mice. Panel **A**: Amino acid concentrations in plasma (μM; mean ± SEM). Panel **B**: Amino-acid fluxes (mean ± SEM) of Ass-Con and Ass-KO/I mice are expressed in arbitrary units (arterio-venous difference in concentration * relative flow across the respective organs). Production is indicated in black and consumption in red numbers. Statistical evaluation was performed by ANOVA. At this age, no significant differences in intestinal and hepatic amino-acid metabolism were observed between Ass-Con and Ass-KO/I mice (all P > 0.1). For a comparison of ND14 Ass-KO/I and ND35 Ass-Con, see Figure 4C.
