# Supplementary figures and images for: Hepatic Adaptation Compensates Inactivation of Intestinal Arginine Biosynthesis in Suckling Mice
Source: PLoS One. 2013 Jun 13;8(6):e67021. doi: 10.1371/journal.pone.0067021 (PMC3681768; doi:10.1371/journal.pone.0067021)

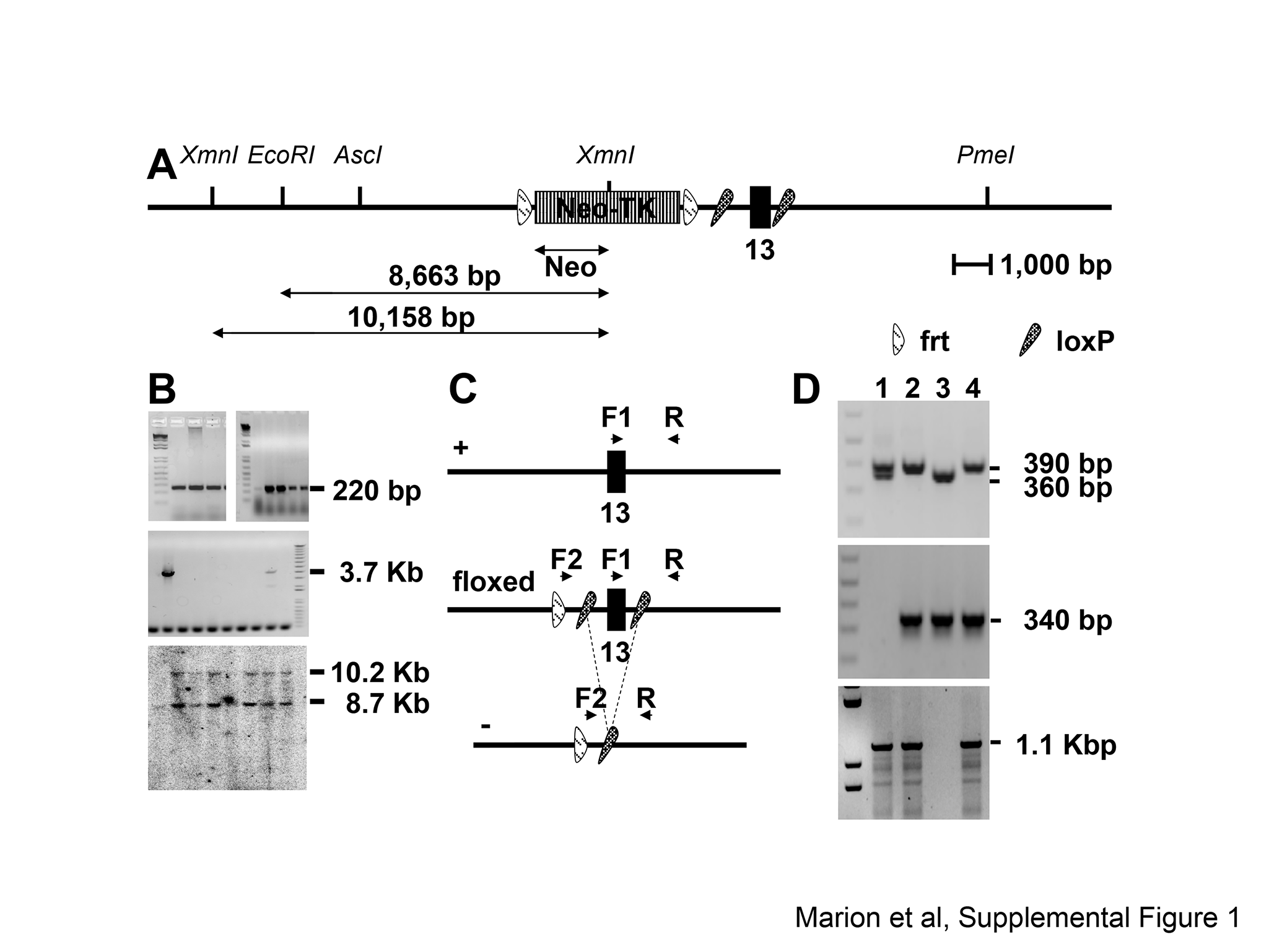

Supplement: Figure S1 — Panel A: Schematic representation of the Ass-targeting construct (AscI – PmeI). The AscI and PmeI sites flanking the construct (14.38 Kb) were introduced by PCR. The XmnI and EcoRI sites upstream from the recombination arms of the targeting construct were used for Southern-blot characterization. Panel B: Identification of successfully targeted ES cells. Top subpanel: PCR amplification of vector sequences downstream of AscI (left) or upstream of PmeI (right) identify clones that carried random integrations of the targeting vector. Middle subpanel: Long PCR to demonstrate homologous recombination on 3’ side with primer in loxP site and primer downstream of PmeI, yielding a band of 3.7kb. Bottom subpanel: Southern blot of ES cell DNA with proper 3’ homologous recombination after digestion with XmnI and EcoRI. Two fragments of the expected size (8.663kb and 10.158kb) were detected with the internal Neo probe. Panel C: Wild-type (Ass+) exon-13 allele, floxed (Assfl) allele, and Ass-deficient (Ass‑) allele, together with remnant frt and loxP sites. F1, F2, and R1 indicate the position of the primers used to identify these genotypes (for sequences, see Table S1). Panel D: Mouse genotyping. Top subpanel: The F1 and R1 primer pair yield a band of 360 bp for the Ass + allele and 390 bp for the Ass fl allele. Middle subpanel: The F2 and R1 primer pair yield a band of 340 bp for the Ass ‑ allele. Bottom subpanel: The presence of the VilCre construct was detected with primer pair Vil-F and Vil-R, giving a band of ~1,100 bp. [file pone.0067021.s001.tif]

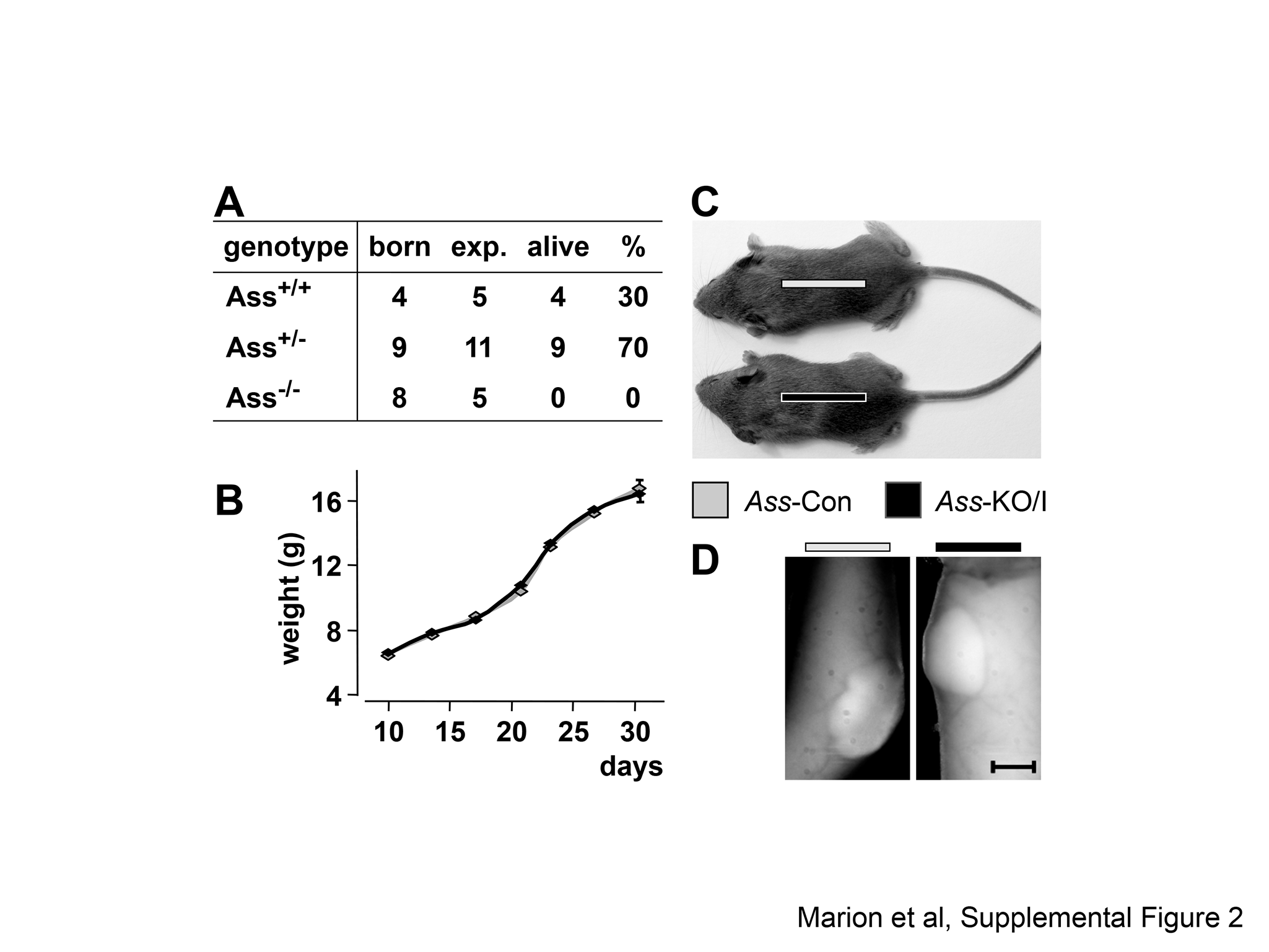

Supplement: Figure S2 — Panel A: Cre-mediated homozygous germ-line elimination of exon 13 causes neonatal death, as was previously shown for constitutive elimination of exon 4 [44,45]. Panels B–D: Ass-KO/I mice grow normally during the first 4 postnatal weeks (B, N = 8 for the wild type and the knockout mice), have normal hair growth (ND14; C) and normal development of Peyer’s patches in the small intestine (ND14; D). Light gray bars indicate Ass-Con and black symbols Ass-KO/I mice. [file pone.0067021.s002.tif]
