# Supplementary material for: Hepatic Adaptation Compensates Inactivation of Intestinal Arginine Biosynthesis in Suckling Mice
Source: PLoS One. 2013 Jun 13;8(6):e67021. doi: 10.1371/journal.pone.0067021 (PMC3681768; doi:10.1371/journal.pone.0067021)
Supplement: Table S1 [file pone.0067021.s003.docx]

**Table S1: Primer sequences, annealing temperatures and expected PCR-product lengths**

| primer | sequence | °C | length |
| --- | --- | --- | --- |
| Ass-F1 (exon13) | 5’gaggaagggacagtggggtg3’ | 60 | *Ass^+^*: F1→ ←R1: 360bp  *Ass^fl^*: F1→ loxP ←R1: 390bp  *Ass^fl^*: F2→loxP-exon13-loxP←R1: 1,000bp  *Ass^-^*: F2→loxP←R1: 340bp |
| Ass-R1 (intron13) | 5’actgctcagggcacgcatgtg3’ |  |  |
| Ass-F2 (*) | 5’tctagaactagtggatcacctcag3’ |  |  |
| Vil-F | 5’tgcctggcctaaagctcac3’ | 62 | 1,100 bp |
| Vil-R | 5’cgacagtatcggcctcagg3’ |  |  |

***:** new DNA sequence inserted between the frt and loxP sites
