# Supplementary material for: Hepatic Adaptation Compensates Inactivation of Intestinal Arginine Biosynthesis in Suckling Mice
Source: PLoS One. 2013 Jun 13;8(6):e67021. doi: 10.1371/journal.pone.0067021 (PMC3681768; doi:10.1371/journal.pone.0067021)
Supplement: Table S2 [file pone.0067021.s004.docx]

**Table S2.** **Genes tested for expression in the small intestine and liver of ND14 *Ass*-Con and *Ass*-KO/I mice**

|  | Gene symbol | Gene Name |  | RefSeq |  | Amplicon Length |
| --- | --- | --- | --- | --- | --- | --- |
| EC3.5.1.2 | *Gls1* | glutaminase |  | NM_001081081.2\|NM_001113383.1 |  | 114 |
| EC3.5.1.2 | *Gls2* | glutaminase 2 (liver, mitochondrial) |  | NM_001033264.3 |  | 118 |
| EC6.3.5.5 | *Cps1* | carbamoyl-phosphate synthetase 1 |  | NM_001080809.1 |  | 69 |
| EC2.3.1.1 | *Nags* | N-acetylglutamate synthase |  | NM_178053.4\|NM_145829.1 |  | 62 |
| EC6.3.4.5 | *Ass1* | argininosuccinate synthetase 1 |  | NM_007494.3 |  | 125 |
| EC4.3.2.1 | *Asl* | argininosuccinate lyase |  | NM_133768.4 |  | 51 |
| EC2.6.1.1 | *Got1* | glutamate oxaloacetate transaminase 1 |  | NM_010324.2 |  | 87 |
| EC2.6.1.1 | *Got2* | glutamate oxaloacetate transaminase 2 |  | NM_010325.2 |  | 75 |
| EC2.6.1.2 | *Gpt1* | glutamic pyruvic transaminase |  | NM_182805.2 |  | 73 |
| EC2.6.1.2 | *Gpt2* | glutamic pyruvate transaminase (alanine aminotransferase) 2 |  | NM_173866.3 |  | 64 |
| EC1.4.1.3 | *Glud1* | glutamate dehydrogenase 1 |  | NM_008133.4 |  | 55 |
| EC3.5.3.1 | *Arg1* | arginase type I |  | NM_007482.3 |  | 65 |
| EC3.5.3.1 | *Arg2* | arginase type II |  | NM_009705.3 |  | 69 |
| EC2.6.1.13 | *Oat* | ornithine aminotransferase |  | NM_016978.2 |  | 81 |
| EC1.5.1.12 | *Aldh18a1* | aldehyde dehydrogenase 18 family, member A1 |  | NM_153554.1\|NM_019698.1 |  | 65 |
| EC1.5.1.2 | *Pycr1* | pyrroline-5-carboxylate reductase 1 |  | NM_144795.3 |  | 82 |
| EC2.1.4.1 | *Gatm* | glycine amidinotransferase (L-arginine:glycine amidinotransferase) |  | NM_025961.5 |  | 70 |
| EC2.1.1.2 | *Gamt* | guanidinoacetate methyltransferase |  | NM_010255.3 |  | 105 |
| EC4.2.1.1 | *Ca5a* | carbonic anhydrase 5a |  | NM_007608.2 |  | 87 |
|  | *Slc1a2* | solute carrier family 1 (glial high affinity glutamate transporter), member 2 |  | NM_001077515.2\|NM_001077514.3\|NM_011393.2 (2 transcript variants) |  | 64 |
|  | *Slc1a4* | solute carrier family 1 (glutamate/neutral amino acid transporter), member 4 |  | NM_018861.3 |  | 75 |
|  | *Slc1a5* | solute carrier family 1 (neutral amino acid transporter), member 5 |  | NM_009201.2 |  | 59 |
|  |  |  |  |  |  |  |
|  | *Slc25a15* | solute carrier family 25 (mitochondrial carrier ornithine transporter), member 15 |  | NM_181325.4 |  | 63 |
|  | *Slc25a2* | solute carrier family 25 (mitochondrial carrier, ornithine transporter) member 2 |  | NM_001159275.1 |  | 107 |
|  | *Slc25a12* | solute carrier family 25 (mitochondrial carrier, Aralar), member 12 |  | NM_172436.3 |  | 70 |
|  | *Slc25a11* | solute carrier family 25 (mitochondrial carrier oxoglutarate carrier), member 11 |  | NM_024211.2 |  | 97 |
|  | *Slc3a2* | solute carrier family 3 (activators of dibasic and neutral amino acid transport), member 2 |  | NM_001161413.1\|NM_008577.4 (2 transcript variants) |  | 92 |
|  | *Slc3a1* | solute carrier family 3, member 1 |  | NM_009205.2 |  | 88 |
|  | *Slc7a7* | solute carrier family 7 (cationic amino acid transporter, y+ system), member 7 |  | NM_011405.3 |  | 100 |
|  | *Slc7a6* | solute carrier family 7 (cationic amino acid transporter, y+ system), member 6 |  | NM_178798.3 |  | 116 |
|  | *Slc7a5* | solute carrier family 7 (cationic amino acid transporter, y+ system), member 5 |  | NM_011404.3 |  | 86 |
|  | *Slc7a8* | solute carrier family 7 (cationic amino acid transporter, y+ system), member 8 |  | NM_016972.2 |  | 98 |
|  | *Slc7a10* | solute carrier family 7 (cationic amino acid transporter, y+ system), member 10 |  | NM_017394.4 |  | 121 |
|  | *Slc7a9* | solute carrier family 7 (cationic amino acid transporter, y+ system), member 9 |  | NM_021291.2 |  | 55 |
|  | *Slc7a14* | solute carrier family 7 (cationic amino acid transporter, y+ system), member 14 |  | NM_172861.3 |  | 68 |
|  | *Slc7a1* | solute carrier family 7 (cationic amino acid transporter, y+ system), member 1 |  | NM_007513.4 |  | 65 |
|  | *Slc7a2* | solute carrier family 7 (cationic amino acid transporter, y+ system), member 2 |  | NM_001044740.1\|NM_007514.3 (2 transcript variants) |  |  |
|  | *Slc7a3* | solute carrier family 7 (cationic amino acid transporter, y+ system), member 3 |  | NM_007515.2 |  | 80 |
|  | *Slc7a4* | solute carrier family 7 (cationic amino acid transporter, y+ system), member 4 |  | NM_144852.3 |  | 67 |
|  | *Slc42a2* | Rhesus blood group-associated B glycoprotein |  | NM_021375.3 |  | 64 |
|  | *Slc38a2* | solute carrier family 38, member 2 |  | NM_175121.3 |  | 61 |
|  | *Slc38a4* | solute carrier family 38, member 4 |  | NM_027052.3 |  | 69 |
|  | *Slc38a3* | solute carrier family 38, member 3 |  | NM_023805.2 |  | 86 |
|  | *Slc38a5* | solute carrier family 38, member 5 |  | NM_172479.2 |  | 73 |
| EC 1.2.1.12 | *Gapdh* | glyceraldehyde-3-phosphate dehydrogenase |  | NM_008084.2 |  | 107 |
|  | *Actb* | actin, beta |  | NM_007393.3 |  | 115 |
