# Supplementary material for: Hepatic Adaptation Compensates Inactivation of Intestinal Arginine Biosynthesis in Suckling Mice
Source: PLoS One. 2013 Jun 13;8(6):e67021. doi: 10.1371/journal.pone.0067021 (PMC3681768; doi:10.1371/journal.pone.0067021)
Supplement: Table S3 — Panel A: Amino acid concentrations in plasma (μM; mean ± SEM). Panel B: Amino-acid fluxes (mean ± SEM) of Ass-Con and Ass-KO/I mice are expressed in arbitrary units (arterio-venous difference in concentration * relative flow across the respective organs). Production is indicated in black and consumption in red numbers. Statistical evaluation was performed by ANOVA (see Figure 4B). [file pone.0067021.s005.docx]

**Table S3: Plasma amino-acid concentrations and fluxes in ND14 Ass-Con and Ass-KO/I mice**. Panel A: Amino acid concentrations in plasma (μM; mean ± SEM). **Panel B:** Amino-acid fluxes (mean ± SEM) of Ass-Con and Ass-KO/I mice are expressed in arbitrary units (arterio-venous difference in concentration * relative flow across the respective organs). Production is indicated in black and consumption in red numbers. Statistical evaluation was performed by ANOVA (see Figure 4B).

| **A** |  | **Glu** | **Asn** | **Ser** | **Gln** | **His** | **Gly** | **Thr** | **Cit** | **Arg** | **Ala** | **Tau** | **Tyr** | **Val** | **Met** | **Ile** | **Phe** | **Trp** | **Leu** | **Orn** | **Lys** | **Sum** |
| --- | --- | --- | --- | --- | --- | --- | --- | --- | --- | --- | --- | --- | --- | --- | --- | --- | --- | --- | --- | --- | --- | --- |
| aorta | Con | 86±9 | 67±5 | 256±21 | 504±12 | 76±4 | 516±28 | 258±16 | 94±6 | 232±13 | 339±42 | 541±45 | 142±9 | 215±13 | 86±5 | 101±5 | 70±4 | 87±4 | 142±6 | 131±11 | 504±25 | 4447±159 |
|  | KO/I | 146±20 | 79±12 | 258±33 | 556±65 | 90±9 | 569±39 | 273±36 | 140±21 | 232±11 | 514±54 | 870±41 | 135±17 | 281±57 | 104±16 | 124±20 | 73±8 | 87±5 | 173±30 | 161±38 | 536±86 | 5518±552 |
| portal | Con | 138±13 | 112±7 | 401±16 | 408±25 | 110±5 | 676±25 | 363±17 | 114±7 | 342±16 | 688±37 | 772±49 | 164±7 | 270±14 | 111±5 | 141±4 | 94±3 | 96±3 | 187±6 | 233±17 | 647±18 | 6023±93 |
| vein | KO/I | 168±20 | 131±14 | 478±64 | 584±90 | 134±21 | 601±47 | 440±49 | 223±24 | 351±26 | 901±125 | 880±52 | 189±16 | 403±54 | 129±13 | 176±16 | 101±9 | 103±6 | 248±27 | 281±42 | 745±61 | 7284±586 |
| hepatic | Con | 189±14 | 127±14 | 478±45 | 547±48 | 136±9 | 684±51 | 400±21 | 132±12 | 356±27 | 646±41 | 866±79 | 204±16 | 337±29 | 127±6 | 175±10 | 112±9 | 114±5 | 238±17 | 341±31 | 709±42 | 6918±270 |
| vein | KO/I | 127±12 | 122±10 | 453±36 | 482±21 | 130±19 | 585±36 | 350±28 | 213±15 | 239±18 | 629±67 | 740±130 | 220±19 | 277±20 | 113±6 | 131±10 | 103±8 | 94±4 | 182±17 | 249±31 | 681±38 | 6118±311 |
| renal | Con | 149±9 | 94±4 | 331±17 | 531±26 | 105±6 | 572±39 | 301±13 | 60 ±3 | 263±14 | 470±29 | 560±31 | 185±10 | 229±11 | 113±9 | 97±5 | 73±3 | 94±3 | 134±8 | 152±8 | 646±29 | 5158±177 |
| vein | KO/I | 130±12 | 102±11 | 335±49 | 614±73 | 104±9 | 519±47 | 344±48 | 124±16 | 318±25 | 538±61 | 668±47 | 162±24 | 355±64 | 90±12 | 146±20 | 78±9 | 84±5 | 201±32 | 219±41 | 691±110 | 5914±554 |

| **B** |  | **Glu** | **Asn** | **Ser** | **Gln** | **His** | **Gly** | **Thr** | **Cit** | **Arg** | **Ala** | **Tau** | **Tyr** | **Val** | **Met** | **Ile** | **Phe** | **Trp** | **Leu** | **Orn** | **Lys** | **Sum** |
| --- | --- | --- | --- | --- | --- | --- | --- | --- | --- | --- | --- | --- | --- | --- | --- | --- | --- | --- | --- | --- | --- | --- |
| splanch | Con | 103±17 | 60±15 | 222±49 | 43±49 | 60±10 | 167±58 | 143±27 | 37±13 | 124±30 | 307±59 | 325±91 | 63±19 | 122±32 | 40±8 | 74±11 | 42±9 | 27±6 | 97±18 | 227±33 | 205±49 | 2471±313 |
| nic area | KO/I | -19±23 | 43±16 | 195±48 | -74±68 | 40±21 | 16±53 | 77±46 | 74±26 | 7±21 | 116±87 | -131±136 | 85±25 | -4±61 | 9±17 | 6±22 | 30±11 | 7±6 | 9±34 | 87±49 | 145±94 | 799±477 |
| PDV | Con | 46±14 | 40±8 | 128±23 | -85±25 | 29±6 | 140±33 | 93±20 | 18±8 | 97±18 | 308±50 | 204±59 | 20±10 | 49±17 | 22±6 | 36±5 | 21±4 | 8±4 | 40±8 | 90±17 | 126±27 | 1387±162 |
|  | KO/I | 19±25 | 46±16 | 194±63 | 25±98 | 39±20 | 28±54 | 147±54 | 73±28 | 104±25 | 341±120 | 9±58 | 48±21 | 108±69 | 22±19 | 45±23 | 25±10 | 14±7 | 66±35 | 106±49 | 184±93 | 1595±602 |
| liver | Con | 57±19 | 20±16 | 95±48 | 128±53 | 30±10 | 27±57 | 50±27 | 20±13 | 27±31 | -1±56 | 121±92 | 43±18 | 73±32 | 18±8 | 38±11 | 21±9 | 19±6 | 56±18 | 137±35 | 79±46 | 1084±289 |
|  | KO/I | -39±24 | -3±17 | 1±71 | -99±90 | 1±27 | -12±58 | -71±55 | 0±28 | -98±31 | -225±137 | -139±140 | 38±25 | -111±58 | -13±15 | -39±20 | 4±12 | -7±8 | -57±32 | -18±51 | -39±75 | -795±640 |
| kidney | Con | 21±4 | 9±2 | 25±9 | 9±10 | 10±3 | 19±16 | 15±7 | -11±2 | 10±6 | 45±17 | 7±19 | 15±5 | 5±3 | 9±2 | -1±2 | 1±2 | 2±2 | -3±3 | 7±4 | 38±13 | 242±81 |
|  | KO/I | -6±8 | 8±6 | 26±20 | 20±33 | 5±4 | -17±21 | 24±20 | -5±9 | 29±9 | 8±28 | -69±21 | 9±10 | 25±29 | -5±7 | 8±10 | 2±4 | -1±2 | 9±15 | 20±19 | 24±40 | 82±175 |
