# Supplementary material for: Hepatic Adaptation Compensates Inactivation of Intestinal Arginine Biosynthesis in Suckling Mice
Source: PLoS One. 2013 Jun 13;8(6):e67021. doi: 10.1371/journal.pone.0067021 (PMC3681768; doi:10.1371/journal.pone.0067021)
Supplement: Table S4 — Panel A: Amino acid concentrations in plasma (μM; mean ± SEM). Panel B: Amino-acid fluxes (mean ± SEM) of Ass-Con and Ass-KO/I mice are expressed in arbitrary units (arterio-venous difference in concentration * relative flow across the respective organs). Production is indicated in black and consumption in red numbers. Statistical evaluation was performed by ANOVA. At this age, no significant differences in intestinal and hepatic amino-acid metabolism were observed between Ass-Con and Ass-KO/I mice (all P > 0.1). For a comparison of ND14 Ass-KO/I and ND35 Ass-Con, see Figure 4C. [file pone.0067021.s006.docx]

**Table S4: Plasma amino-acid concentrations and fluxes in ND35 Ass-Con and Ass-KO/I mice. Panel A:** Amino acid concentrations in plasma (μM; mean ± SEM). **Panel B:** Amino-acid fluxes (mean ± SEM) of Ass-Con and Ass-KO/I mice are expressed in arbitrary units (arterio-venous difference in concentration * relative flow across the respective organs). Production is indicated in black and consumption in red numbers. Statistical evaluation was performed by ANOVA. At this age, no significant differences in intestinal and hepatic amino-acid metabolism were observed between Ass-Con and Ass-KO/I mice (all P > 0.1). For a comparison of ND14 Ass-KO/I and ND35 Ass-Con, see Figure 4C.

| **A** |  | **Glu** | **Asn** | **Gln** | **His** | **Thr** | **Cit** | **Arg** | **Ala** | **Tau** | **Tyr** | **Val** | **Met** | **Ile** | **Phe** | **Trp** | **Leu** | **Orn** | **Lys** | **Sum** |
| --- | --- | --- | --- | --- | --- | --- | --- | --- | --- | --- | --- | --- | --- | --- | --- | --- | --- | --- | --- | --- |
| Aorta | Con | 57±6 | 63±6 | 577±26 | 40±4 | 212±15 | 74±7 | 162±14 | 867±87 | 216±19 | 281±25 | 318±17 | 72±5 | 183±13 | 69±4 | 272±18 | 214±16 | 32±11 | 497±36 | 4278±239 |
|  | KO/I | 50±6 | 65±6 | 549±25 | 34±4 | 201±13 | 74±3 | 149±11 | 883±70 | 197±15 | 297±22 | 283±17 | 66±4 | 159±11 | 67±5 | 252±15 | 185±13 | 49±10 | 437±35 | 4096±218 |
| portal vein | Con | 115±9 | 224±23 | 397±44 | 124±21 | 352±34 | 122±8 | 212±17 | 1924±151 | 277±31 | 303±30 | 398±32 | 89±5 | 254±26 | 103±11 | 344±24 | 298±33 | 109±41 | 653±37 | 6269±490 |
|  | KO/I | 140±20 | 243±31 | 456±32 | 178±53 | 422±60 | 140±9 | 238±23 | 2288±220 | 354±49 | 307±21 | 428±38 | 97±6 | 279±32 | 128±16 | 362±28 | 326±39 | 333±153 | 689±62 | 7332±759 |
| hepatic vein | Con | 19±5 | 89±17 | 498±23 | 64±18 | 267±35 | 110±10 | 166±24 | 1078±181 | 227±43 | 342±35 | 377±34 | 77±8 | 239±29 | 93±11 | 302±31 | 278±38 | 99±45 | 583±67 | 4900±580 |
|  | KO/I | 33±7 | 83±10 | 516±33 | 76±15 | 248±24 | 114±6 | 136±14 | 1017±107 | 212±19 | 322±32 | 329±19 | 68±4 | 204±16 | 88±6 | 255±22 | 245±19 | 172±35 | 485±46 | 4547±294 |

| **B** |  | **Glu** | **Asn** | **Gln** | **His** | **Thr** | **Cit** | **Arg** | **Ala** | **Tau** | **Tyr** | **Val** | **Met** | **Ile** | **Phe** | **Trp** | **Leu** | **Orn** | **Lys** | **Sum** |
| --- | --- | --- | --- | --- | --- | --- | --- | --- | --- | --- | --- | --- | --- | --- | --- | --- | --- | --- | --- | --- |
| Splanch | Con | -37±8 | 21±18 | -48±50 | 22±21 | 43±43 | 31±14 | 0±30 | 169±208 | 0±49 | 52±46 | 47±43 | 3±10 | 47±34 | 19±13 | 22±39 | 54±44 | 68±53 | 69±81 | 478±662 |
| nic area | KO/I | -16±9 | 18±11 | -33±41 | 42±15 | 47±27 | 40±7 | -13±18 | 135±127 | 15±24 | 24±39 | 46±25 | 2±6 | 44±19 | 21±8 | 3±26 | 60±23 | 123±36 | 48±58 | 451±366 |
| PDV | Con | 49±9 | 137±20 | -153±43 | 71±18 | 119±32 | 41±9 | 42±19 | 898±148 | 52±31 | 19±33 | 68±31 | 14±6 | 61±25 | 29±10 | 61±26 | 72±31 | 66±36 | 133±44 | 1692±463 |
|  | KO/I | 77±17 | 152±26 | -79±34 | 0±45 | 188±52 | 55±8 | 75±22 | 1195±196 | 134±43 | 8±26 | 123±35 | 26±6 | 102±29 | 51±14 | 94±27 | 120±35 | 241±131 | 214±60 | 2751±671 |
| Liver | Con | -86±10 | -115±27 | 105±59 | -49±28 | -76±51 | -10±15 | -43±31 | -729±237 | -52±54 | 33±48 | -21±50 | -11±10 | -14±40 | -10±17 | -39±41 | -18±51 | 2±65 | -64±82 | -1215±770 |
|  | KO/I | -93±20 | -134±30 | 46±45 | -80±51 | -140±60 | -15±11 | -88±26 | -1060±231 | -118±49 | 16±39 | -77±40 | -24±7 | -57±33 | -31±16 | -91±34 | -60±41 | -118±146 | -166±75 | -2299±764 |
